# Intensity of cervical inflammatory reaction as a risk factor for recurrence of carcinoma of the uterine cervix in stages IB and IIA

**DOI:** 10.1590/S1516-31802007000400008

**Published:** 2007-07-01

**Authors:** José Humberto Tavares Guerreiro Fregnani, Fernando Augusto Soares, Pablo Roberto Novik, Ademar Lopes, Maria do Rosário Dias de Oliveira Latorre

**Keywords:** Uterine cervical neoplasms, Recurrence, Survival, Inflammation, Lymphatic metastasis, Neoplasm invasiveness, Neoplasias do colo do útero, Recidiva, Sobrevivência, Inflamação, Metástase linfática, Invasividade neoplásica

## Abstract

**CONTEXT AND OBJECTIVE:**

Inflammatory reaction intensity has been indicated as a possible recurrence risk factor in carcinoma of the uterine cervix. Some authors observed greater risk with weak inflammatory reaction, while others described the opposite. This study aimed to evaluate risk factors for initial-stage uterine cervix carcinoma recurrence (IB and IIA), considering inflammatory reaction intensity.

**DESIGN AND SETTING:**

Retrospective cohort at Hospital do Câncer A. C. Camargo.

**METHODS:**

289 patients with diagnosed uterine cervix carcinoma (stages IB and IIA) who underwent radical surgery between 1980 and 1999 were studied. Data were collected from medical records. Histological sections from tumors and lymph nodes could be reviewed in 247 cases. Five-year disease-free survival rates were calculated using the Kaplan-Meier method and curves were compared using the log-rank test. Cox's proportional-hazards model was used for multivariate analysis. Recurrence risk was estimated using hazard ratios (HR).

**RESULTS:**

Forty-three recurrences were found. Multivariate analysis identified the following independent recurrence risk factors: number of metastatic pelvic lymph nodes (one lymph node: HR = 3.3 [1.3-8.3]; two or three: HR = 5.3 [1.5-18.6]; four or more: HR = 7.6 [1.7-33.2]), tumor invasion depth (deepest third: HR = 2.1 [1.1-4.1]) and inflammatory reaction intensity in the uterine cervix (absent or slight: HR = 2.5 [1.1-5.7]).

**CONCLUSION:**

This study identified that absent or slight inflammatory reaction was an independent risk factor for recurrence. The other risk factors were the number of metastatic pelvic lymph nodes and invasion of the deepest third of the uterine cervix.

## RESUMO

**CONTEXTO E OBJETIVO:**

A intensidade da reação inflamatória é citada como possível fator de risco para recorrência do carcinoma do colo do útero (CCU). Alguns autores observaram aumento do risco com reação inflamatória fraca, enquanto outros descreveram o oposto. Este estudo procurou avaliar os fatores de risco para recorrência do CCU em estádios iniciais (IB e IIA), considerando na análise a reação inflamatória.

**TIPO DE ESTUDO E LOCAL:**

Coorte retrospectiva, no Centro de Tratamento e Pesquisa Hospital A. C. Camargo (Hospital do Câncer).

**MÉTODOS:**

Estudaram-se 289 pacientes com diagnóstico de CCU (estádios IB e IIA) submetidas à cirurgia radical entre 1980 e 1999. Realizou-se a coleta de dados nos prontuários. Os cortes histológicos dos tumores e dos linfonodos foram revistos em 247 casos. As taxas de sobrevida livre de doença em cinco anos foram calculadas pelo método de Kaplan-Meier e as curvas comparadas pelo teste de log-rank. Para a análise multivariada empregou-se o modelo de riscos proporcionais de Cox. A estimativa do risco de recorrência foi o hazard ratio (HR).

**RESULTADOS:**

Registraram-se 43 recorrências. A análise multivariada identificou os seguintes fatores de risco independentes para recorrência: número de linfonodos pélvicos metastáticos (1 linfonodo: HR = 3,3 [1,3-8,3]; 2 ou 3 linfonodos: HR = 5,3 [1,5-18,6]; 4 ou mais linfonodos: HR=7,6 [1,7 – 33,2]), profundidade de invasão do tumor (terço profundo: HR = 2,1 [1,1-4,1]) e intensidade da reação inflamatória no colo do útero (ausente ou leve: HR = 2,5 [1,1-5,7]).

**CONCLUSÃO:**

Este estudo identificou a reação inflamatória ausente ou de intensidade leve como fatores de risco independentes para recorrência. Os outros fatores de risco foram o número de linfonodos pélvicos metastáticos e a invasão do terço profundo do colo do útero.

## INTRODUCTION

In developed countries, carcinoma of the uterine cervix represents around 4% of all tumors among women and is the sixth most frequent type of cancer in the population. In developing countries, it is the most common or second most common type of cancer among women, corresponding to 15% of tumors.^1^

The treatment is dictated by the clinical staging that is recommended by the International Federation of Gynecology and Obstetrics (Fédération Internationale de Gynécologie et d’Obstétrique, FIGO).^2^ The initial stages IB and IIA can be treated by means of Piver-Rutledge class III radical hysterectomy,^3^ in association with pelvic lymphnodectomy, or by means of radiotherapy alone.^4^ In the more advanced stages (IIB to IVB), radiotherapy presents better results than does surgery.^5^ Around 20% of the patients with carcinoma of the uterine cervix at its initial stages (IB and IIA) who have been adequately treated by radical surgery develop disease recurrence.^6^ In 90% of such cases, the recurrence occurs within two years.^7^ The reason for this recurrence is persistence of the disease at microscopic scale locoregionally (pelvis) and/or at a distance, in an organ (for example, in the lungs, liver or bones).

The literature describes a variety of risk factors for recurrence of carcinoma of the uterine cervix in its initial stages, such as the presence of metastases in lymph nodes, tumor size, depth of invasion into the cervical stroma, invasion of the parametrium, histological type, degree of cell differentiation, blood capillary embolization and lymphatic capillary embolization, among others.^8^ The intensity of the inflammatory reaction in the uterine cervix consequent to carcinoma has been indicated as a risk factor for recurrence. If on the one hand some authors have observed increased risk of recurrence when the inflammatory reaction was weak,^9-21^ others have described discordant results.^22-25^

Reinthaller et al. evaluated 158 women with carcinoma of the uterine cervix in stages IA to IIB and observed that the women with a severe inflammatory reaction in the uterine cervix presented lower recurrence rates, independent of the histological type.^14^ Kainz et al. identified the intensity of the cervical inflammatory reaction as a risk factor for recurrence, in multivariate analysis. Absent or moderate inflammatory reaction implied a reduction in disease-free survival.^15^ In a study on 73 cases of carcinoma of the uterine cervix in stage IB, Bethwaite et al. noted a significant association between low density of T lymphocytes in the uterine cervix and disease recurrence.^16^ Chao et al. evaluated 83 patients with carcinoma of the uterine cervix (stage IIB) who underwent radical hysterectomy and found that, in the cases in which the intensity of the inflammatory reaction in the cervix was slight, recurrence was significantly more frequent (50.0% versus 16.9%).^18^ Horn et al. evaluated patients with carcinoma of the uterine cervix in its initial stage who underwent surgical treatment and found that absence of peritumoral inflammatory reaction was associated with greater risk of recurrence.^20^

The intensity of the inflammatory reaction in the uterine cervix is considered in some scoring systems to be a risk factor for the recurrence of carcinoma of the uterine cervix, as in the Malignancy Grading System^10^ and the Partial Index.^11^ In these scoring systems, and also in others that are derived from them,^12,13,17,19^ the absence of inflammatory reaction or a slight reaction is implicated in greater recurrence risk. However, neither of these scoring systems is based on multivariate analysis to determine the intensity of the inflammatory reaction as a risk factor for recurrence.

A weak inflammatory reaction in the uterine cervix denotes failure of the immune system. By synthesizing immunomodulators, neoplastic cells are able to escape from the immunity surveillance mechanisms, thereby promoting depression of the local cell immunity. A variety of mediators involved in immunodepression are produced by neoplastic cells, and the following are prominent: transforming growth factor-beta (TGF-β), interleukin IL-4, interleukin IL-6, interleukin IL-10, macrophage chemotactic protein-1 (MCP-1), macrophage deactivating factor (MDF), suppressive E-receptor (SER), immunosuppressive acidic protein (IAP), prostaglandin PGE2 and protein p15E, among others.^26,27^ Recently, it has been reported that certain metalloproteinases not only degrade the extracellular matrix but also are implicated in the immunosuppression promoted by the tumor.^28^

## OBJECTIVE

To evaluate the risk factors for recurrence of carcinoma of the uterine cervix in its initial stages (IB and IIA), considering the intensity of the inflammatory reaction in the uterine cervix in this analysis.

## METHODS

This was a retrospective cohort study in which patients diagnosed with carcinoma of the uterine cervix who underwent radical surgical treatment at the Treatment and Research Center of Hospital do Câncer A. C. Camargo between 1980 and 1999 were evaluated. The inclusion criteria were: 1) Histological proof of invasive carcinoma of the uterine cervix; 2) Clinical staging IB and IIA, according to the criteria established by FIGO; 3) Absence of any previous treatment; 4) Surgical treatment performed by means of Piver-Rutledge class II or III radical hysterectomy^3^ and pelvic lymphnodectomy.

The medical records were consulted and information was collected on a specific form for sociodemographic, clinical, histopathological, therapeutic and follow-up data. The histological sections from the tumors and lymph nodes (stained using hematoxylin-eosin, HE) were reviewed in 247 cases. In 42 cases, it was not possible to conduct the review because of the poor quality of the slides or because the slides could not be located in the files of the Department of Pathology.

The sample was composed of 289 women, whose ages ranged from 18 to 72 years (mean = 44.4 years; standard deviation, SD = 10.0 years; median = 44.0 years). Most of them were white (75.8%) or brown-skinned (16.6%), married (76.2%) and of low educational level (17.6% were illiterate and 71.3% had only done elementary education). Most of the patients were staged clinically as IB (90.7%) and the others as IIA.

The radical hysterectomy method most frequently utilized was type III (97.9%) and the remaining hysterectomies were type II. The mean number of lymph nodes dissected during the surgery was 19.4 (SD = 8.6; median = 19.0), with a range from 1 to 60. Involved surgical margins were observed in 3.8% of the patients (11 cases).

Postoperative radiotherapy was administered to 118 patients (40.8%). Of these, in 39 cases the indication for complementary treatment was because of metastases in the pelvic lymph nodes. In the other cases, although the lymph nodes did not present metastases shown by the HE method, at least one of the following events was observed: tumor lesions larger than 4 cm; moderately or poorly differentiated tumors; presence of deep invasion into the cervical stroma; lymphatic capillary embolization; blood capillary embolization; involved or narrow surgical margins; and involvement of the parametrium.

Teletherapy was utilized in association with vaginal brachytherapy in 89 cases, vaginal brachytherapy alone was utilized in 20 cases, and teletherapy alone was utilized in nine cases. In two cases, the adjuvant radiotherapy was utilized in association with chemotherapy (cisplatin 40 mg/m^2^), both in the year 1999. The adjuvant teletherapy dose utilized ranged from 540 to 6040 cGy, with a median of 4500 cGy (mean = 4612 cGy; SD = 831 cGy).

The patients were followed up for a mean of 103.7 months, ranging from seven days to 259.5 months (SD = 63.2 months; median = 102.7 months). Sixty-three patients (21.8%) were lost to follow-up, and their mean follow-up was 47.8 months (SD = 44.2 months; median = 38.6 months), ranging from 15 days to 208.9 months.

The information collected was stored in a computerized database and analyzed by means of the Statistical Package for the Social Sciences (SPSS)^®^ software, version 10.0. Five-year disease-free survival rates were calculated using the Kaplan-Meier method, and the survival curves were compared using the log-rank test. Multivariate analysis was performed using Cox's proportional-hazards model, and the recurrence risk was estimated using hazard ratios (HR). The modeling technique was the stepwise forward selection method, and the significance level was set at 5.0%.

This study formed part of a larger research project within the postgraduate program at Fundação Antônio Prudente. It was submitted in advance to, and approved by, the Research Ethics Committee of the Treatment and Research Center of Hospital do Câncer A. C. Camargo (approval no. 354/01). Because this was a retrospective study, there was no need to draw up an informed consent statement.

## RESULTS

Over the study period, 43 recurrences (14.9%) were recorded, distributed thus: 22 within the pelvic region, 12 at a distance, seven simultaneously pelvic and at a distance and two at unknown locations. The mean length of time until recurrence was 29.3 months (SD = 24.9 months; median = 21.9 months), ranging from 2.4 to 99.0 months. The five-year disease-free survival rate for the population studied was 86.3%.

Univariate analysis showed that, among the sociodemographic and clinical variables, only a number of previous gestations greater than four (p = 0.020) was associated with recurrence (Table 1). Among the histopathological variables, associations were observed between recurrence and the number of metastatic pelvic lymph nodes (p = 0.001); the depth of invasion of the tumor, stratified into thirds (p = 0.008); and the intensity of the inflammatory reaction in the uterine cervix (p = 0.009). Among the treatment-related variables, none showed any association with recurrence (Table 2).

**Table 1.** Five-year disease-free survival rate according to sociodemographic and clinical variables. Hospital A. C. Camargo (Hospital do Câncer), 1980-1999

Variable	Category	n*	Five-year disease-free survival (%)	p
**Age**	Up to 45 years	159	87.5	0.252
	More than 45 years	128	84.8	
**Skin color**	White	217	87.3	0.429
	Non-white	70	83.2	
**Education level**	Up to completion of elementary education†	230	86.2	0.929
	High school or university-level	29	85.5	
**Number of previous gestations**	Up to 4	140	90.1	0.020
	More than 4	144	82.4	
**Menopause**	No	209	86.7	0.247
	Yes	75	84.7	
**Clinical stage**	IB	200	86.8	0.917
	IIA	63	87.9	

* Cases with unknown figures were excluded from the analysis;

† Includes illiterate patients.

**Table 2.** Five-year disease-free survival rate according to histopathological and treatment-related variables. Hospital A. C. Camargo (Hospital do Câncer), 1980-1999

Variable	Category	n*	Five-year disease-free survival (%)	p
**Histological type**	Epidermoid	204	85.9	0.714
	Adenocarcinoma	35	87.9	
	Adenoscamous	5	74.0	
**Histological grade**	1	44	92.4	0.161
	2	124	82.6	
	3	78	83.9	
**Mitotic index**	Up to 10 cga	60	87.1	0.174
	11 to 20 cga	74	79.9	
	More than 20 cga	110	89.7	
**Tumor size**	Up to 2 cm	58	78.6	0.238
	2.1 to 4.0 cm	96	88.8	
	More than 4.0 cm	33	86.5	
**Depth of invasion into the uterine cervix**	Superficial and middle thirds	159	89.2	0.008
	Deepest third	85	80.3	
**Capillary embolization (blood and/or lymphatic)**	No	125	87.3	0.271
	Yes	120	84.7	
**Number of metastatic pelvic lymph nodes**	0	240	88.8	0.001
	1	22	68.8	
	2 or 3	17	79.9	
	4 or more	8	71.4	
**Perineural invasion**	No	215	86.6	0.190
	Yes	30	82.5	
**Invasion of the parametrium**	No	112	83.8	0.234
	Yes	7	100.0	
**Invasion of the uterine body**	No	218	84.9	0.493
	Yes	34	87.1	
**Intensity of the inflammatory reaction in the uterine cervix**	Absent or slight	141	81.4	0.009
	Moderate or severe	105	92.6	
**Tumor necrosis**	Absent or slight	190	85.8	0.804
	Moderate or severe	56	87.0	
**Decade of treatment**	1980s	157	81.9	0.163
	1990s	130	91.1	
**Type of radical hysterectomy**	II	6	83.3	0.847
	III	281	86.4	
**Number of lymph nodes dissected**	Less than 20	152	89.1	0.187
	20 or more	134	83.0	
**Surgical margins**	Free	247	85.7	0.799
	Involved	11	90.9	
**Postoperative radiotherapy**	No	165	83.2	0.311
	Yes	118	90.2	

* Cases with unknown figures were excluded from the analysis.

The multivariate model identified the following independent risk factors for recurrence: number of metastatic pelvic lymph nodes (one lymph node: hazard ratio, HR = 3.3; two or three lymph nodes: HR = 5.3; four or more lymph nodes: HR = 7.6); depth of tumor invasion into the uterine cervix (deepest third: HR = 2.1); and intensity of the inflammatory reaction in the uterine cervix (absent or slight: HR = 2.5) (Table 3).

**Table 3.** Independent variables associated with recurrence that were identified using Cox's proportional-hazards model (adjusted for adjuvant radiotherapy and decade of treatment)

Variable	Category	HR (unadjusted)	HR (adjusted)	95% CI (adjusted HR)
**Number of metastatic pelvic lymph nodes**	0	1.0	1.0	Ref.
	1	3.1	3.3	1.3-8.3
	2 or 3	3.4	5.3	1.5-18.6
	4 or more	3.6	7.6	1.7-33.2
**Depth of invasion into the uterine cervix**	Superficial or middle third	1.0	1.0	Ref.
	Deepest third	2.4	2.1	1.1-4.1
**Intensity of the inflammatory reaction in the uterine cervix**	Moderate or severe	1.0	1.0	Ref.
	Absent or slight	2.8	2.5	1.1-5.7

HR = hazard ratio; 95% CI = 95% confidence interval. Ref= reference categry.

## DISCUSSION

In the present sample, the independent variables associated with recurrence were the number of pelvic lymph nodes involved in metastases, the depth of invasion into the cervical stroma and the intensity of the inflammatory response in the uterine cervix. Of these, only the latter is not habitually cited in the literature as a risk factor for recurrence.

However, there are authors who have been unable to demonstrate any difference in the risk of recurrence, in relation to the intensity of the inflammatory reaction in the uterine cervix.^22-24^ Others have found results that are the opposite of what is present above.^25^ Studying prognostic factors in 196 cases of carcinoma of the uterine cervix in stages IB and IIA without metastases in the lymph nodes, Samlal et al. found that the risk of recurrence was around 2.5 times greater in cases with severe inflammatory reaction in the uterine cervix.^25^

Lack of standardization of the criteria utilized for defining the intensity of the inflammatory reaction is probably responsible for this difference in results. In the present study, the fact that a single pathologist reviewed the tumor slides reduced the bias produced by variation between observers. Nonetheless, the reproducibility of the results found here is debatable, because what may correspond to slight inflammatory reaction in one pathologist's opinion may represent a moderate reaction according to another pathologist. There is a need to develop criteria for assessing the intensity of the inflammatory reaction that are less subjective, thereby enabling better reproduction of the results in future studies.

The presence of metastases in pelvic lymph nodes is the most important histopathological factor in carcinoma of the uterine cervix for overall, cancer-specific and disease-free survival.^19,29^ Nevertheless, the simple presence or absence of metastases in the lymph nodes is not the only factor implicated in recurrence. The number of lymph nodes involved has also been described as a risk factor for recurrence, and progressively shorter disease-free survival with increasing numbers of lymph node metastases has been noted.^17,30-34^ In the present sample, the recurrence risk became progressively greater with increasing numbers of metastatic pelvic lymph nodes.

The depth of tumor invasion into the uterine cervical stroma showed a close relationship with the recurrence of carcinoma of the uterine cervix, in the present study. This result is in agreement with published findings.^17,35-37^ Samlal et al. demonstrated that the depth of tumor invasion is a risk factor for recurrence even in patients without lymph node metastases.^25^

Differing from what is described in the literature, some variables in the present study did not show any association with recurrence risk, not even in the univariate analysis. This was, for example, found with regard to capillary embolization, which has been strongly associated with the presence of regional lymph node metastases in cases of carcinoma of the uterine cervix, and also with recurrence.^29^ Identification of emboli inside capillaries is not always an easy task, since the retraction of the peritumoral stroma that takes place in histological sections may make it difficult to identify capillary invasion in slides stained using hematoxylin-eosin.^29^ It is possible that this technical difficulty may have created a bias. Another variable that did not show any association with recurrence was tumor size, which has traditionally been described as a risk factor for recurrence. Since surgical specimens are not stored in the Department of Pathology of the Treatment and Research Center of Hospital do Câncer A. C. Camargo, the tumor measurements were obtained from the anatomopathological reports that were available in the medical records. Since these anatomopathological examinations were not performed by the same person, the tumor measurement technique probably varied, which created the bias that is responsible for this result.

The classical criteria for indicating postoperative radiotherapy in cases of carcinoma of the uterine cervix are the presence of lymph node metastases, tumor lesions larger than 4 cm, moderately or poorly differentiated tumors, presence of deep stromal invasion, lymphatic capillary embolization, blood capillary embolization, involved or narrow surgical margins, and involvement of the lateral cervical ligaments (parametrium).^38^ It needs to be considered at this stage whether patients who had not presented any of these risk factors for recurrence but whose anatomopathological examination demonstrated a weak inflammatory reaction in the uterine cervix should undergo postoperative radiotherapy. The answer is that they probably should receive radiotherapy, but this procedure should not be adopted as the routine in cancer treatment centers until the matter has been duly assessed by means of prospective studies, considering that adjuvant radiotherapy is not a harmless procedure.^38^ It will only be possible to assess the real benefit of including inflammatory reaction in the indications for postoperative radiotherapy through prospective studies that are appropriately set up for this purpose.

## CONCLUSION

This study observed that patients with absent or slight inflammatory reaction presented 2.5 times greater risk of recurrence than did patients with a moderate or severe reaction. Other risk factors implicated in recurrence were the number of metastatic pelvic lymph nodes and invasion of the deepest third of the uterine cervix.
